# Investigation of the *SLC22A23* gene in laryngeal squamous cell carcinoma

**DOI:** 10.1186/s12885-018-4381-y

**Published:** 2018-04-27

**Authors:** Seda Ekizoglu, Didem Seven, Turgut Ulutin, Jalal Guliyev, Nur Buyru

**Affiliations:** 1 Cerrahpasa Medical Faculty, Department of Medical Biology, Istanbul University, Kocamustafapasa, 34098 Istanbul, Turkey; 2 Cerrahpasa Medical Faculty, Department of Otorhinolaryngology, Istanbul University, Istanbul, Turkey

**Keywords:** Laryngeal cancer, GeneFishing, SLC22A23, Expression, Genotyping

## Abstract

**Background:**

Laryngeal squamous cell carcinoma (LSCC) is the second most common cancer of the head and neck. In order to identify differentially expressed genes which may have a role in LSCC carcinogenesis, we performed GeneFishing Assay. One of the differentially expressed genes was the *SLC22A23* (solute carrier family 22, member 23) gene.

SLC22A23 belongs to a family of organic ion transporters that are responsible for the absorption or excretion of many drugs, xenobiotics and endogenous compounds in a variety of tissues. SLC22A23 is expressed in a various tissues but no substrates or functions have been identified for it. Although the exact function is unknown, single nucleotide polymorphisms (SNPs) which are located in *SLC22A23* gene were associated with inflammatory bowel disease (IBD), endometriosis-related infertility and the clearance of antipsychotic drugs. On the other hand *SLC22A23* is identified as a prognostic gene to predict the recurrence of triple-negative breast cancer.

**Methods:**

To understand the role of the *SLC22A23* gene in laryngeal carcinogenesis, we investigated its mRNA expression level in laryngeal tumor tissue and adjacent non-cancerous tissue samples obtained from 83 patients by quantitative real-time PCR. To understand the association between SNPs in *SLC22A23* and LSCC, selected genetic variations (rs4959235, rs6923667, rs9503518) were genotyped.

**Results:**

We found that *SLC22A23* expression was increased in 46 of 83 tumor tissues (55.4%) and was decreased in 30 of 83 (36.1%) tumor tissues compared to normal tissues. 77.2% of patients were homozygote for genotype rs9503518-AA and they most frequently had histological grade 2 and 3 tumors. We also found that rs9503518-AA genotype is associated with increased *SLC22A23* expression.

**Conclusions:**

Our results indicate that *SLC22A23* may play a role in the development of laryngeal cancer.

## Background

Laryngeal squamous cell carcinoma (LSCC) is the second most common cancer of the head and neck [1]. It has been proposed that LSCC is a complex disease caused by the interaction of genetic and environmental factors. Smoking, high alcohol consumption and human papillomavirus infections have been considered as the major environmental factors [2, 3]. Although, early detection and diagnosis of LSCC can greatly increase the success of treatment by surgery, chemotherapy and radiothearapy, the 5-year survival rates vary between 40 and 80% depending on the anatomical location [4]. Therefore, a better understanding of the mechanisms underlying LSCC is of great importance and several studies have addressed the identification of target genes involved in LSCC pathogenesis.

Solute carrier (SLC) transporters comprise one of the two membrane transporters with more than 300 members which have been divided into 52 families [5, 6]. The main functions of these proteins is to transfer a wide range of substrates such as amino acids, lipids, inorganic ions, peptides, saccharides, metal ions, proteins, xenobiotics and drugs [7, 8]. Therefore, the effect of each transporter on the cell behaviour depends on the type of the molecule it transports. While some of the members such as organic anion transporters are involved in chemoresistance, some may play a role in cell survival and cell cycle progression because of their function in nutrient transportation [9]. One of the known functions of the SLC proteins is to facilitate the uptake of nutrients and removal of metabolites. It is well known that cancer cells need extra metabolic requirements during rapid cell cycles. Accumulating evidence supports that many SLC transporters are up-regulated in various cancers to supply the increasing demand of the tumor cells [9, 10]. SLC22A23 (solute carrier family 22, member 23) belongs to the SLC family of organic ion transporters that are responsible for the uptake or excretion of many compounds including drugs, toxins and endogenous metabolites in a variety of tissues [11]. SLC22A23 is expressed in various tissues but no substrates or functions have yet been identified for it [12].

Single nucleotide polymorphisms (SNPs) are variations in individual nucleotides which occur within a gene or in a regulatory region near a gene. They may affect the gene’s function or may have predict an individual’s response to certain drugs, susceptibility to environmental factors and risk of developing particular diseases. SNPs also affect the gene expression rates by changing the nucleotide sequence in the transcription factor bindig domain or the sequence of non-coding RNA binding sites. Several SNPs have been identified in the *SLC22A23* locus previously [13–16]. Therefore, in this study we aimed to investigate the expression levels and probable role of the *SLC22A23* gene SNPs in LSCC.

## Methods

### Samples

A total of 83 patients diagnosed with LSCC were included in this study. Fresh tumors and matching non-cancerous tissue samples were obtained from patients undergoing surgery in the Department of Otorhinolaryngology, Cerrahpasa Medical Faculty. 2 ml of venous blood was collected into EDTA-containing tubes from all patients. There were 80 men (96.4%) and 3 women (3.6%). The mean age at diagnosis was 59 ± 9 years. The clinical characteristics, including stage, histological type, histological grade, smoking status, age and gender are shown in Table 1.

**Table 1:** Clinicopathological characteristics of patients

Parameters	Variable	n (%)
Clinical stage	Early stage (I+ II)	8 (9.6)
	Advanced stage (III+ IV)	74 (89.2)
	Unknown	1 (1.2)
Histology	Squamous cell carcinoma (SCC)	79 (95.2)
	Non-SCC	3 (3.6)
	Unknown	1 (1.2)
Histological grade	Grade 1	2 (2.4)
	Grade 2	35 (42.2)
	Grade 3	32 (38.6)
	Grade 4	7 (8.4)
	Unknown	7 (8.4)
Smoking	Smoker	69 (83.1)
	Non-smoker	12 (14.5)
	Unknown	2 (2.4)
Gender	Female	3 (3.6)
	Male	80 (96.4)
Age	≤50	13 (15.7)
	> 50	69 (83.1)
	Unknown	1 (1.2)

The study was approved by the Cerrahpasa Medical Faculty Ethics Committee (Approval number: 83045809/604.01/02-235,918), and has been performed in accordance with the ethical standarts laid down in the 2013 Declaration of Helsinki. Signed informed consent was obtained from all patients.

### Identification of differentially expressed genes (DEGs) by GeneFishing

#### RNA isolation and GeneFishing reverse transcription

Total RNA was isolated from both tumors and adjacent non-cancerous tissues of 4 patients using the miRCURY RNA Isolation Kit (Exiqon, Vedbaek, Denmark) according to the manufacturer’s instructions. First strand cDNA was prepared from 3 μg of total RNA and reverse transcription was carried out for 90 min at 42 °C and 2 min at 94 °C in a final volume of 20 μl containing 1 μM dT-ACP1 (provided in the GeneFishing DEG Premix Kit, Seegene, Seoul, Korea), 1xRT buffer (Invitrogen, Carlsbad, CA, USA), 0.5 mM dNTP, 20 U RNase inhibitor (BIOMATIK, Wilmington, DE, USA) and 200 U M-MLV reverse transcriptase (Invitrogen, Carsbad, CA, USA). First strand cDNA was diluted by adding 80 μl of DNase-free water.

#### ACP-based GeneFishing polymerase chain reaction

GeneFishing PCRs were performed using a primer set consisting of 20 different arbitrary ACPs (Annealing Control Primers) provided in the GeneFishing DEG Premix Kit (Seegene, Seoul, Korea). The reaction conditions were: diluted first-strand cDNA (50 ng), 0.5 μM arbitrary ACP (one of the arbitrary ACPs), 0.5 μM dT-ACP2 and 1xSeeAmp ACP master mix in a 20 μl final volume. PCR was performed at 94 °C for 5 min, 50 °C for 3 min, 72 °C for 1 min, followed by 40 cycles of 94 °C for 40 s, 65 °C for 40 s and 72 °C for 40 s and a final step for 5 min at 72 °C. The amplified PCR products were separated on 2% agarose gels and the differentially expressed bands were purified from the gels using the Zymoclean Gel DNA Recovery Kit (Zymo Research, Irvine, CA, USA).

### Cloning and sequencing

Purified PCR products were directly cloned into the pCR™4-TOPO vector using the TOPO TA Cloning Kit for Sequencing (Invitrogen, Carlsbad, CA, USA). Following the cloning reaction, the pCR™4-TOPO construct was transformed into competent *E. coli* (One Shot TOP 10) cells according to the One Shot chemical transformation protocol provided in the kit. *E. coli* cells were cultured overnight at 37 °C in LB (Luria-Bertani) agar plates containing 50 μg/ml kanamycin. 2-6 colonies were taken and cultured overnight at 37 °C in LB medium containing 50 μg/ml kanamycin. For identification of the inserted PCR product, the plasmid DNA was isolated using the PureLink Quick Plasmid Miniprep Kit (Invitrogen, Carlsbad, CA, USA) and sequenced on an ABI Prism 3100-Avant™ Genetic Analyzer (Applied Biosystems, Foster City, CA, USA) using the ABI Prism BigDye Terminator v3.1 Cycle Sequencing Kit (Applied Biosystems, Foster City, CA, USA). Sequences were analyzed by searching for similarities using the Basic Local Alignment Search Tool (BLAST) program.

### Quantitative real time polymerase chain reaction (qRT-PCR) analysis of *SLC22A23*

Total RNA was isolated from 83 tumors and adjacent non-cancerous tissues using the PureLink RNA Mini Kit (Ambion, Carlsbad, CA, USA). cDNA was synthesized from 400 ng of total RNA using the RevertAid First-Strand cDNA Synthesis Kit (Thermo Scientific, Waltham, MA, USA).

Expression levels of the *SLC22A23* gene were analyzed by qRT-PCR using the LightCycler 480-II system (Roche Diagnostics, Mannheim, Germany). PCR was performed in a final volume of 15 μl containing 1× master PCR mix (SolGent, Daejeon, South Korea) with EvaGreen (Biotium, Fremont, CA, USA), 600 nM gene-specific primers, nuclease free water and cDNA. The sequences of the primers are shown in Table 2. The PCR amplification protocol was an initial denaturation of 15 min at 95 °C, 40 cycles of amplification at 95 °C for 15 s, 59 °C for 30 s, and 72 °C for 30 s followed by a cooling step of 10 s at 50 °C. The reference gene used for normalization was *Beta-2-microglobulin (B2M)* and relative mRNA levels were calculated by the comparative 2^-ΔΔCt^ method [17].

**Table 2:** Primer sequences used for qRT-PCR

Gene	Primer	Sequence
*SLC22A23*	Forward	5’-ACCCCGACGGTGATAAGGTGT-3′
	Reverse	5’-TCTGGTTGTGCAGCTCGATGAT-3’
*B2M*	Forward	5’-CTCGCGCTACTCTCTCTTTCTGG-3’
	Reverse	5’-GCTTACATGTCTCGATCCCACTTAA-3’

### Genotyping

Genomic DNA was isolated from blood using the High Pure PCR Template Preparation Kit (Roche Diagnostics, Mannheim, Germany) and was kept at − 80 °C until use. The SNPs rs9503518, rs4959235 and rs6923667 within the human *SLC22A23* gene were genotyped using TaqMan SNP Genotyping Assays (Assay ID C__25960793_20, C__27912010_10, C__29004073_10) (Applied Biosystems, Foster City, CA, USA) and the Applied Biosystems 7500 Fast Real-Time PCR System. PCRs were performed in a final reaction volume of 20 μl per well containing 1× TaqMan Genotyping Master Mix (Applied Biosystems, Foster City, CA, USA), 1× SNP TaqMan SNP Genotyping Assay (Applied Biosystems, Foster City, CA, USA) and 20 ng DNA. The reaction conditions included an initial step of 1 min at 60 °C, an enzyme activation step of 10 min at 95 °C and 40 cycles at 95 °C for 15 s and 60 °C for 1 min. Allelic discrimination was determined using the 7500 Fast Real-Time PCR software version 2.3 and FAM and VIC fluorescence probes. The dye used as the passive reference was ROX.

### Statistical analysis

Statistical analyses were performed using IBM SPSS Statistics 20 software (IBM Corp., Armonk, NY, USA). Wilcoxon test and Pearson’s chi-square test are used to calculate *p* values. *p* < 0.05 was considered statistically significant.

## Results

### Identification of differentially expressed genes

To identify Differentially Expressed Genes (DEGs) in LSCC, we compared the mRNA expression profiles of the tumor tissues with those of normal tissues using ACP-based GeneFishing PCR with a combination of 20 arbitrary primers and two anchored oligo (dT) primers (dT-ACP1 and dT-ACP2). The analysis was performed with 4 pairs of tumor and normal tissues.

Twenty-seven DEGs were identified, including 15 down-regulated and 12 up-regulated DEGs in tumor tissue compared with normal tissue. Among these 27 DEGs, 12 DEGs were isolated, cloned, sequenced and searched in the GenBank.

**Fig. 1:**
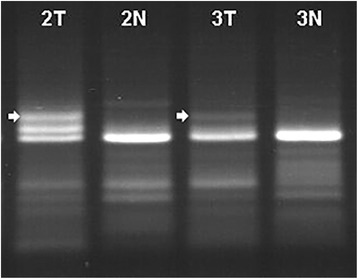
GeneFishing PCR Result*.* PCR products corresponding to the *SLC22A23* gene are indicated by arrows (T: Tumor, N: Normal)

We identified the *SLC22A23* gene by sequence analysis of one of the up-regulated DEGs by homology searching using the Basic Local Alignment Search Tool (BLAST) program. GeneFishig PCR results observed on an agarose gel for *SLC22A23* are shown in Fig. 1.

### Confirmation of the expression pattern of *SLC22A23* by real-time PCR

We examined the altered expression level of the *SLC22A23* gene using Real-Time PCR in 83 tumor samples and adjacent non-cancerous tissue samples. We observed increased *SLC22A23* mRNA expression in 46 of 83 tumor tissues (55.4%) and decreased expression in 30 tissues (36.1%) when compared to their normal counterparts. No change was detected in 7 samples. The 2^-ΔΔCt^ levels were 1.55 and 1 for the tumor and the normal tissue samples, respectively (Table 3). Statistically, significant upregulation of the *SLC22A23* mRNA was observed in laryngeal tumor tissues (*p* = 0.001). No significant correlation was found between *SLC22A23* expression and clinicopathological parameters such as the clinical stage (*p* = 0.329), histology (*p* = 0.067), sex (*p* = 0.286), age (*p* = 0.482), histological grade (*p* = 0.649) and smoking status (p = 0,977).

**Table 3:** Expression values of the *SLC22A23* gene in tumors and normal tissues

	*SLC22A23* Ct (Median)	*B2M* Ct (Median)	ΔCt (Median)	ΔΔCt	2^-ΔΔCt^	*p* ^a^
Tumor	26.7	21.7	5	−0.6	1.55	0.014
Normal	27.6	22.3	5.3	0	1	

^a^Statistical analyses were performed using the Wilcoxon test

### Genotyping of the *SLC22A23* polymorphisms

Genotyping of the *SLC22A23* rs9503518, rs4959235 and rs6923667 polymorphisms was carried out by real-time PCR allelic discrimination analysis. Genotype and allele frequencies for each SNP are shown in Table 4.

**Table 4:** Genotypes and allele frequencies

Variation number	Genotype	n	Genotype Frequency	Allele	Allele Frequency
rs9503518	AA	61	0.772	A	0.842
	GG	7	0.089	G	0.158
	AG	11	0.139		
rs4959235	CC	58	0.841	C	0.920
	TT	0	0	T	0.080
	CT	11	0.159		
rs6923667	CC	28	0.384	C	0.603
	TT	13	0.178	T	0.397
	CT	32	0.438		

We observed that 77.2% of patients carried the homozygote AA-genotype for rs9503518, and 43.8% of patients carried the heterozygote CT-genotype for rs6923667. 84.1% of patients were homozygous for rs4959235-CC and 15.9% were heterozygous for rs4959235-CT but we didn’t observe rs4959235-TT homozygotes in our study group. We didn’t find any association between the rs4959235-CC/CT, rs6923667-CC/CT/TT genotypes and clinicopathological parameters such as the clinical stage, histology, sex, age, histological grade and smoking status. But we observed that patients who were homozygous for rs9503518-AA most frequently had histological grade 2 and 3 tumors and the association was statistiacally significant (Table 5).

**Table 5:** Association of rs9503518 with histological grade

		rs9503518			
		AA n (%)	GG n (%)	AG n (%)	*p* ^a^
Histological Grade	Grade 1	0 (0)	2 (2.5)	0 (0)	0.002
	Grade 2	24 (30.4)	3 (3.8)	6 (7.6)	
	Grade 3	26 (32.9)	2 (2.5)	3 (3.8)	
	Grade 4	6 (7.6)	0 (0)	1 (1.3)	
	Unknown	5 (6.3)	0 (0)	1 (1.3)	

^a^Statistical analyses were performed using the Pearson’s chi-square test

Moreover, we investigated if SNPs of the *SLC22A23* gene play a role in the expression level of the gene and found that 52.2% of homozygote patients for genotype rs9503518-AA had increased *SLC22A23* gene expression (Table 6). The association between rs9503518-AA and *SLC22A23* expression level was statistically significant (*p* = 0.046). No significant association was found between the *SLC22A23* gene expression and rs4959235-CC/CT and rs6923667-CC/CT/TT genotypes.

**Table 6:** Association between the *SLC22A23* polymorphisms and gene expression

Variation number	Genotype	*SLC22A23* Gene Expression			
		No change n (%)	Decreased n (%)	Increased n (%)	*p* ^a^
rs9503518	A/A	6 (7.6)	23 (29.1)	32 (40.5)	0.046
	G/G	0 (0)	0 (0)	7 (8.9)	
	A/G	0 (0)	7 (8.9)	4 (5.1)	
rs4959235	C/C	5 (7.2)	19 (27.5)	34 (49.3)	0.360
	C/T	1 (1.4)	6 (8.7)	4 (5.8)	
rs6923667	C/C	1 (1.4)	12 (16.4)	15 (20.5)	0.556
	T/T	1 (1.4)	4 (5.5)	8 (11)	
	C/T	5 (6.8)	11 (15.1)	16 (21.9)	

^a^Statistical analyses were performed using the Pearson’s chi-square test

## Discussion

SLC transporters is one the largest membrane transporter families with more than 300 members and 52 subfamilies [5, 6]. They play a major role in the transport of many different charged and uncharged organic molecules in addition to inorganic ions [7, 8]. The SLC22 subfamily is responsible for the transport of organic ions and has been clustered in three different subgroups based on function and sequence homology such as organic cation transporters (OCTs), organic anion transporters (OATs) and organic zwitterion transporters (OCTNs) [18, 19].

Most of the OATs generally facilitate the movement of organic anions into the epithelial cells and are known as influx transporters [20]. Depending on their location OATs function in the uptake, reabsorption and excretion of drugs, nutrients and metabolites [18]. The best investigated OAT is *OAT1 (SLC22A6)* which has been cloned in 1996 as a kidney transporter [21]. Although OATs are also present in all barrier epithelia of the body, in liver, plasenta and brain; most of the SLC22A investigations have focused on the kidney. Accumulating evidence suggests that OATs are up-regulated in malignant tumors probably to supply the increased nutritional demand of the tumor cells. On the other hand, many members of the solute carriers have been associated with the uptake, distribution and excretion of several drugs [22–26]. It has been reported that renal drug excretion in proximal tubules is mediated by SLC22 family transporters [27, 28]. Shinatsar et al. investigated mRNA expression levels of some members of the SLCA22A family in renal cell carcinoma cell lines and reported that expression of *SLC22A3* increases the chemosensitivity to some drugs in kidney carcinoma cell lines [29]. Some other members of the SLC22A have been associated with pathological characteristics of the tumor cells. For example, a high level of *SLC22A18* has been associated with the smaller tumor size while lower levels of *SLC22A1* and *SLC22A11* have been associated with angioinvasion in pancreatic ductal adenocarcinoma (PDAC) [30]. Database analysis has also shown that *SLC22A7* expression is associated with multicentric tumor occurence in hepatocellular carcinoma [31]. Depending upon Triple Negative Breast (TNB) cancer prediction and pathway analysis Chen et al. identified 6 genes, one of these being *SLC22A23* [32]. However, detailed information is not available on the *SLC22A23* gene or its substrate. The first analysis of SLC22A23 has been performed by Bennet et al. who isolated the *SLC22A23* gene as a human homolog of the rat organic cation transporter by rapid amplification of cDNA ends (RACE) [12]. Additionally they also analyzed expression of the *SLC22A23* gene in cell lines. Performing functional expression analysis they proposed that SLC22A23 requires additional molecules or co-factors to show functional activity in the membrane transport. So far there is no study in the literature investigating the expression rate of the *SLC22A23* gene in cancer. Therefore, in view of our DEGs results we investigated expression levels of the *SLC22A23* gene in larynx tumor samples and observed up-regulation of the *SLC22A23* mRNA levels in a significant proportion of the tumors.

In recent years, it has been shown that SNPs in the membrane transporter genes may be involved in tumor development and progression as well as in the regulation of drug resistance. For example, SNPs *SLC22A1*, *SLC22A2*, *SLC22A6* and *SLC22A8* have been reported to be implicated in altered drug response [22, 33, 34]. Therefore, we also investigated three SNPs of the *SLC22A23* gene. One of these polymorphisms (rs9503518) has been associated with increased risk of cardiac arythmias. Some other polymorphisms of the *SLC22A23* gene have also been associated with complex diseases that have an inflammatory component such as IBD, endometriosis-related infertility which is an indicator of the transporter activity of the *SLC22A23* gene [13–15]. On the other hand, Aberq et al. attributed the QTc prolongation to the presence of rs4959235 polymorphism in the *SLC22A23* gene [16]. They proposed that rs4959235 mediates the effects of quetiapine via clearence of the drug from the heat or shuttling of the molecules which are involved in cardiac function. In our study group we observed an association between the rs9503518 polymorphism and the histological grade of the tumor. This indicates that *SLC22A23* may function in supplying of the nutritional needs of the cell. However, there is no data in the literature yet about the substrate of this transporter molecule.

## Conclusions

In conclusion, as a preliminary report our results indicate that *SLC22A23* acts as one of the membrane transporters in larynx cancer which warrants further investigation in larynx cancer.

## Abbreviations

ACP: Annealing Control Primer
B2M: Beta-2-microglobulin
BLAST: Basic Local Alignment Search Tool
DEG: Differently expressed gene
EDTA: Ethylenediaminetetraacetic acid
IBD: Inflammatory bowel disease
LB: Luria-Bertani
LSCC: Laryngeal squamous cell carcinoma
OAT: Organic anion transporter
OCT: Organic cation transporter
OCTN: Organic zwitterion transporter
PDAC: Pancreatic ductal adenocarcinoma
qRT-PCR: Quantitative real time polymerase chain reaction
RACE: Rapid Amplification of cDNA Ends
SLC: Solute carrier
SLC22A23: Solute carrier family 22, member 23
SNP: Single nucleotide polymorphism
TNB: Triple negative breast
